# Navigating Combined Laser Modalities Versus Monotherapy for Atrophic Acne and Postsurgical Scar Improvement: A Systematic Review and Network Meta‐Analysis

**DOI:** 10.1111/jocd.71230

**Published:** 2026-10-29

**Authors:** Qiao Yang, Jue Wang, Kai Yang, Guoping Feng, Yan Liu, Chengcheng Li, Yang Liu, Jipeng Song, Linxiumei Guo

**Affiliations:** ^1^ Beijing Jishuitan Hospital Capital Medical University Beijing China

**Keywords:** acne scar, combined laser, laser therapy, network meta‐analysis, postsurgical scar

## Abstract

**Background:**

Combined laser protocols are increasingly used for atrophic acne and postsurgical scars, but no laser‐only network meta‐analysis has assessed these regimens within a focused laser‐versus‐laser framework.

**Objective:**

To map randomized evidence for laser monotherapy and combined‐laser treatment, identify evidence gaps, and generate provisional treatment rankings.

**Methods:**

PubMed, Europe PMC, OpenAlex, and ClinicalTrials.gov were searched from inception through April 2026. Eligible studies were randomized controlled trials comparing at least 2 laser modalities, either as monotherapy or in combination, for atrophic acne or postsurgical scars. Risk of bias was assessed with Cochrane RoB 2.0, and a random‐effects frequentist network meta‐analysis synthesized standardized mean differences and *P*‐scores.

**Results:**

Twenty‐six RCTs enrolling 771 participants across 14 laser nodes were included. Only 17 of 91 possible pairwise comparisons (19%) were supported by direct randomized evidence. The 4 combined‐laser regimens were each evaluated in a single RCT; the 5 trials contributing at least 1 combination‐versus‐monotherapy comparison enrolled 172 participants. The largest trial reported better Goodman‐Baron improvement with PDL plus nonablative fractional laser than with NAFL alone (standardized mean difference, approximately 0.56). In the main connected monotherapy network, picosecond Nd:YAG ranked highest (*P*‐score, 0.858), but certainty was very low. Overall risk of bias was low in 4 studies and showed some concerns in 22.

**Conclusion:**

Evidence for combined‐laser scar treatment remains sparse and insufficient to recommend a specific dual‐laser regimen. This review mainly clarifies current evidence gaps and priorities for future comparative trials.

## 1 Introduction

A high prevalence rate for atrophic acne scars is reported among those with inflammatory acne, accounting for 55%–77% of cases, with moderate to severe deformity in approximately 30%. Postsurgical scars share the same aesthetic and physiological problems, and timely laser treatment could help direct wound healing in the right direction.

Laser treatment promotes scar remodeling through stimulation of dermal renewal, reduction of vascularity, and skin surface smoothing. Commonly employed lasers include ablative fractional lasers like fractional CO_2_ and Er:YAG, non‐ablative fractional lasers, vascular lasers including PDL (pulsed dye laser), and more recently developed picosecond Nd:YAG lasers. The fact that these techniques address different components of the pathophysiology of scars makes combined therapy an attractive approach.

This rationale has encouraged multimodal protocols in routine practice. Early randomized trials suggest potential benefit: Zhou et al. [1] reported greater Goodman–Baron improvement with PDL plus 1565‐nm NAFL than with NAFL alone, and Cohen and Geronemus [2] observed the highest scar‐improvement scores in a combined PDL‐plus‐FCO_2_ arm for post‐Mohs scars. Whether these findings generalize across laser pairings and scar types remains uncertain.

Previous network meta‐analyses have usually combined laser and non‐laser interventions, limiting laser‐specific interpretation. Zhao et al. [3] included PRP, radiofrequency microneedling, and chemical reconstruction alongside laser platforms, a design that broadens network connectivity but is less informative when the clinical question is which laser should be selected.

The review was therefore restricted to comparisons among laser treatments, including dual laser approaches but excluding non‐laser therapies, where applicable. The aims included mapping the literature, identifying gaps in comparisons, and synthesizing quantifiable information for developing provisional rankings in a clinically coherent laser treatment‐only network.

## 2 Methods

### 2.1 Protocol and Registration

This review followed PRISMA 2020 [4] and PRISMA‐NMA guidelines. All aspects of the eligibility criteria, search strategy, data extraction items, risk‐of‐bias assessment, and analytical approach were predetermined in the study protocol and not changed post hoc.

### 2.2 Eligibility Criteria

The criteria for selection of studies for inclusion were based on the PICOS principle. The target population was defined as individuals with clinically diagnosed atrophic acne scars, which included ice pick, boxcar, and rolling types of scars, or those with post‐surgical scarring, independent of location and duration after the initial procedure. In addition, the interventions consisted of any treatments using at least two different lasers combined, either sequentially in a single session or consecutively in successive sessions. Examples of such combinations involved the use of pulsed dye laser (PDL) and fractional CO_2_ (FCO_2_), non‐ablative fractional laser (NAFL) with ablative fractional laser (AFL), among others with Er:YAG, Nd:YAG, picosecond, thulium, or diode lasers. The comparator was defined as any single laser modality used as monotherapy; direct head‐to‐head comparisons between two different monotherapies were also eligible so as to ensure adequate network connectivity for indirect treatment ranking. The primary outcome was clinical scar improvement assessed by validated scoring instruments—specifically the Échelle d'Évaluation Clinique des Cicatrices d'Acné (ECCA), Goodman and Baron Quantitative Scarring Grading System, or Vancouver Scar Scale (VSS)—while secondary outcomes included patient satisfaction scores, the incidence of post‐inflammatory hyperpigmentation (PIH), erythema duration, crusting duration, and pain during treatment. Only randomized controlled trials (RCTs), including split‐face and split‐scar intraindividual designs with documented randomization of treatment allocation, were considered for inclusion.

Studies were excluded if they were nonrandomized; included non‐laser adjuncts; focused exclusively on hypertrophic, keloid, or burn scars; lacked extractable outcome data; were reviews, editorials, conference abstracts, animal studies, or duplicates; or if full English text could not be obtained.

### 2.3 Information Sources and Search Strategy

An exhaustive literature search was performed from inception up until April 2026 in four electronic databases (MEDLINE via PubMed, Europe PMC, OpenAlex, and ClinicalTrials.gov). This was complemented by backward citation searching of identified papers as well as forward citation searching of previous network meta‐analyses that examined partly similar clinical topics. The search was carried out using a Boolean logic approach incorporating three groups of concepts, namely laser‐related MeSH or free‐text terminologies (Group 1), scar‐related patient population terminologies (Group 2), and a specific search filter limited to randomized trials and split‐face design trials only (Group 3). In Group 1, both MeSH and free‐text terms were considered in order to include all forms of lasers, including fractional CO_2_, Er:YAG, pulsed dye, Nd:YAG, picosecond, thulium, and diode lasers, as well as fractional ablative and nonablative lasers and their respective wavelengths. Group 2 comprised the MeSH headings “Cicatrix” and “Acne Vulgaris,” as well as free‐text terms for atrophic, ice‐pick, boxcar, and rolling scars. The publication type code of randomized controlled trials was combined with other free‐text codes relating to randomization, split‐face, half‐face, split‐scar, and intraindividual design. Search strategies tailored to each of the databases (PubMed, Europe PMC, OpenAlex, and ClinicalTrials.gov) are provided in Appendix S1. In addition, all references of previous network meta‐analyses on laser therapy for the treatment of acne scars on the face were manually examined to find any missed studies in the electronic search.

### 2.4 Study Selection

Records were deduplicated and screened in two stages: title/abstract review followed by full‐text assessment. Prespecified PICOS criteria were applied throughout, and reasons for exclusion were documented at full text. Disagreements were resolved by discussion and, when needed, third‐reviewer adjudication. The screening process is summarized in Figure 1.

**FIGURE 1:**
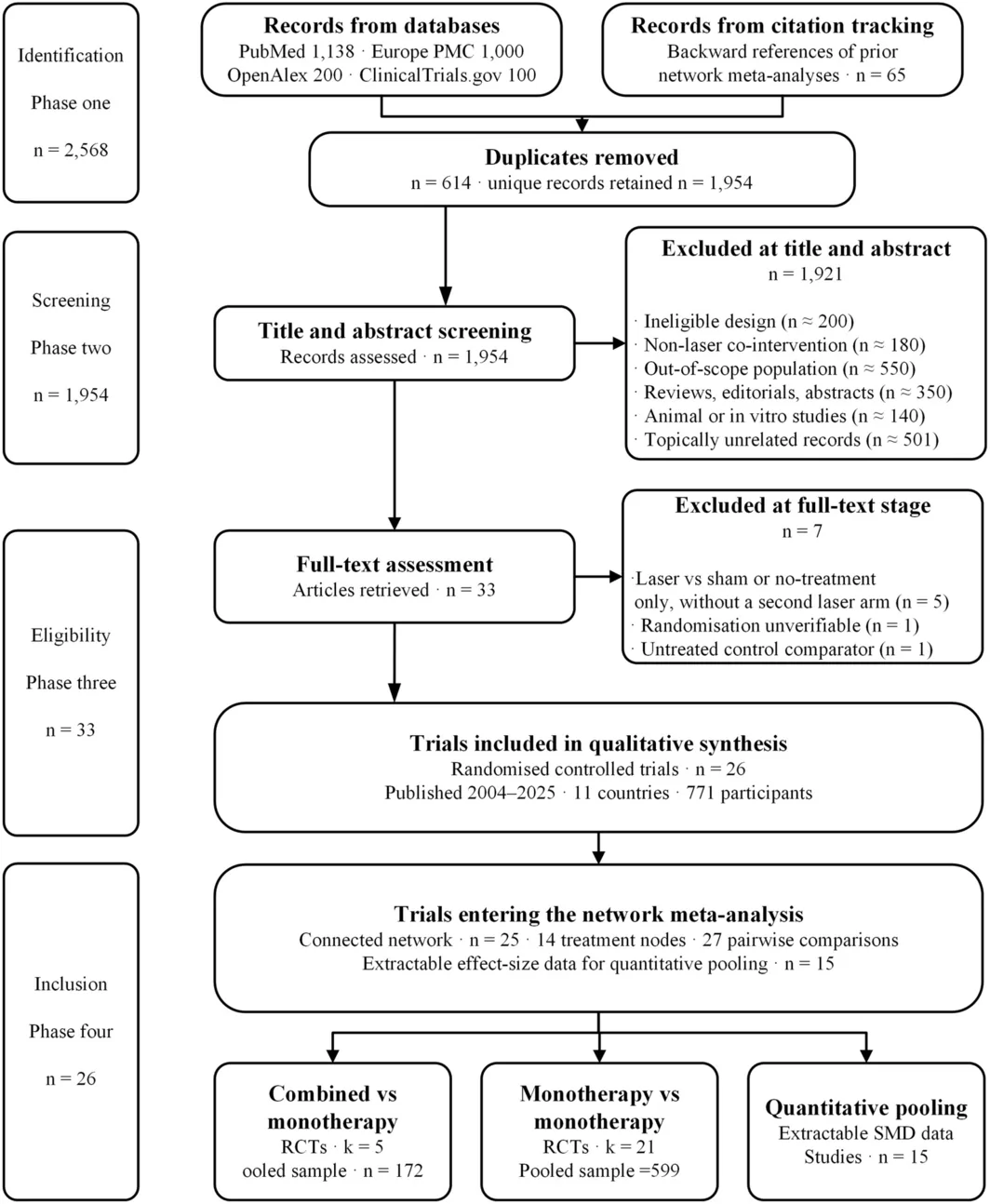
PRISMA 2020 flow diagram. PRISMA 2020 flow diagram showing the identification, screening, eligibility assessment, and inclusion of randomized controlled trials evaluating laser monotherapy and combined‐laser protocols for atrophic acne and postsurgical scars.

### 2.5 Data Extraction

Data extraction used a standardized form covering study design, sample size, scar type, follow‐up, laser parameters, and clinical outcomes. Split‐face and split‐scar trials were extracted at the patient level. Medians were converted using Wan et al. [5], and graphical data were digitized when necessary.

### 2.6 Risk of Bias Assessment

Methodological quality of each included RCT was appraised using the Cochrane Risk of Bias 2.0 (RoB 2.0) tool, which evaluates five domains: the randomization process, deviations from intended interventions, missing outcome data, measurement of the outcome, and selection of reported results. Each domain was judged as carrying a low risk of bias, some concerns, or a high risk of bias, and an overall risk‐of‐bias judgment was derived for every study following the algorithm specified in the RoB 2.0 guidance document. It should be noted that blinding of participants and treatment providers is inherently infeasible in laser trials because of the visible and sensory differences between laser modalities; accordingly, the domain addressing deviations from intended interventions was evaluated with particular attention to whether outcome assessors were blinded rather than whether participants were aware of their assigned treatment.

### 2.7 Statistical Analysis

Network meta‐analysis was performed in R using netmeta with a random‐effects model and REML estimation. Standardized mean differences (Hedges *g*) were used because included studies reported heterogeneous outcome scales. Treatments were ranked with *P*‐scores.

Transitivity was examined by comparing major effect modifiers across direct comparisons. Global inconsistency was assessed with the design‐by‐treatment interaction model proposed by Higgins et al. [6], and local inconsistency with node‐splitting. Small‐study effects were explored with a comparison‐adjusted funnel plot.

Of 26 qualitatively included RCTs, 25 contributed to network geometry. Safra et al. [7] was disconnected because its comparator was no treatment rather than another laser. Fifteen network‐eligible trials provided data suitable for standardized‐mean‐difference pooling; the remaining 10 informed network geometry and evidence‐gap mapping only.

Assessment of small‐study effects and potential publication bias was carried out by visual inspection of a comparison‐adjusted funnel plot ordered according to treatment ranking. The certainty of evidence for the highest‐ranking network comparison, picosecond Nd:YAG versus fractional CO_2_, was further appraised within the Confidence in Network Meta‐Analysis (CINeMA) framework, which evaluates the domains of within‐study bias, reporting bias, indirectness, imprecision, heterogeneity, and incoherence, and classifies the overall certainty of each network estimate as high, moderate, low, or very low.

### 2.8 Evidence Gap Mapping

Given the repositioned objective of this review—to characterize the landscape and identify knowledge gaps in the laser combination evidence base—a pre‐specified evidence gap analysis was performed. A pairwise comparison matrix tabulating the number of available RCTs, the cumulative sample size, and the proportion of studies providing extractable quantitative outcomes was generated for every possible pairing of laser nodes within the network. This matrix was visualized as a heatmap to highlight comparisons supported by robust direct evidence alongside those representing evidence voids for which no randomized data currently exist.

### 2.9 Subgroup and Sensitivity Analyses

A subgroup analysis stratifying studies by scar etiology (acne scars versus postsurgical scars) was planned a priori. A planned additional stratification by study design was ultimately subsumed within the sensitivity analysis framework rather than conducted as a formal subgroup analysis, given that only seven parallel‐group trials were available and their limited network connectivity precluded a stand‐alone subgroup network meta‐analysis. Sensitivity analyses were conducted to assess the robustness of the findings by restricting the network to (a) studies judged as having a low overall risk of bias, (b) split‐face designs only, which mitigate inter‐patient variability, and (c) monotherapy nodes exclusively—thereby removing the sparsely populated combination nodes and evaluating whether the relative ranking of single‐laser modalities remained stable.

## 3 Results

### 3.1 Study Selection

Figure 1 summarizes study selection. Searches identified 2568 records (2503 database records and 65 records from reference tracking). After removal of 614 duplicates, 1954 records underwent title‐and‐abstract screening, and 1921 were excluded. Of 33 full texts assessed, 7 were excluded: 5 compared a laser with sham or no treatment only, 1 lacked verifiable randomization, and 1 used untreated control as the comparator. This left 26 RCTs for qualitative synthesis, 25 for the connected network, and 15 with extractable effect‐size data for quantitative pooling.

### 3.2 Study Characteristics

The 26 included trials were published between 2004 and 2025 and enrolled 771 participants across 11 countries, most commonly South Korea, the United States, China, Thailand, and Egypt. Sample sizes ranged from 12 to 100 (median 20); 19 studies used split‐face or split‐scar designs and 7 used parallel‐group randomization. Nineteen studies addressed atrophic acne scars and 7 examined postsurgical or post‐traumatic scars. Fitzpatrick phototypes were reported in 12 studies, mainly types III–IV. Study characteristics are summarized in Table 1.

**TABLE 1:** Characteristics of the 26 included studies.

Category	Study	Year	Country	Scar type	Design	*N*	Intervention	Comparator	Sessions	Follow‐up	Main outcomes
Combined vs. monotherapy	Cohen et al. [2]	2016	USA	Postsurgical scar	Parallel, multi‐arm RCT	25	PDL+FCO_2_	PDL/FCO_2_	3–4 sessions	3 months	VSS, GER, satisfaction
Combined vs. monotherapy	Safra et al. [7]	2019	Israel	Postsurgical scar	RCT	18	PDL+FCO_2_	No‐treatment control	3 sessions	6 months	VAS scar score
Combined vs. monotherapy	Kim et al. [8]	2009	Korea	Acne scar	Split‐face RCT	20	AFL+NAFL	AFL monotherapy	3 sessions	3 months	Clinical improvement, complications
Combined vs. monotherapy	Zhou et al. [1]	2024	China	Acne scar	Parallel RCT	90	PDL+NAFL	NAFL	1 session	4 weeks	Goodman‐Baron, erythema
Combined vs. monotherapy	Min et al. [9]	2009	Korea	Acne scar	Split‐face RCT	19	Combined 585/1064 nm	Nd:YAG LP	4 sessions	NR	ECCA, satisfaction
Monotherapy vs. monotherapy	Liu et al. [10]	2025	China	Acne scar	Split‐face, evaluator‐blinded RCT	13	NAFL	FCO_2_	Multiple vs. single session	NR	Clinical improvement
Monotherapy vs. monotherapy	Yuan et al. [11]	2025	China	Acne scar	Split‐face RCT	30	Picosecond	FCO_2_	3 sessions	NR	ECCA, physician improvement
Monotherapy vs. monotherapy	Dai et al. [12]	2023	China	Acne scar	Split‐face prospective RCT	31	Picosecond	Er:YAG	4 sessions	20 weeks	ECCA, IGA, satisfaction
Monotherapy vs. monotherapy	Sabry et al. [13]	2022	Egypt	Acne scar	Split‐face, double‐blind RCT	20	Nd:YAG QS	FCO_2_	4 sessions	NR	Clinical improvement
Monotherapy vs. monotherapy	Al‐Dhalimi et al. [14]	2021	Iraq	Acne scar	Split‐face RCT	20	Er:YAG	Nd:YAG LP	3 sessions	3 months	Clinical improvement, satisfaction
Monotherapy vs. monotherapy	Cheng et al. [15]	2021	China	Acne scar	Split‐face, blinded RCT	19	NAFL	FCO_2_	3 sessions	NR	Improvement, erythema, pain
Monotherapy vs. monotherapy	Shi et al. [16]	2021	China	Acne scar	Split‐face, double‐blind RCT	22	Picosecond	NAFL	3 sessions	1 month after final session	ECCA, erythema, pain
Monotherapy vs. monotherapy	Sirithanabadeekul et al. [17]	2021	Thailand	Acne scar	Split‐face RCT	25	Picosecond	FCO_2_	1 session	3 months	ECCA, PIH, satisfaction
Monotherapy vs. monotherapy	Kwon et al. [18]	2020	Korea	Acne scar	Split‐face prospective RCT	25	Picosecond	NAFL	4 sessions	8 weeks after final session	ECCA, IGA, pain, satisfaction
Monotherapy vs. monotherapy	Chitgopeker et al. [19]	2020	USA	Postsurgical/traumatic scar	Split‐scar, blinded RCT	100	FCO_2_	NAFL	3 sessions	NR	Improvement, pain, satisfaction
Monotherapy vs. monotherapy	Shin et al. [20]	2014	Korea	Postsurgical scar	Split‐scar RCT	20	NAFL	AFL/FCO_2_	2 sessions	3 months	Improvement, erythema, pigmentation
Monotherapy vs. monotherapy	Kim et al. [21]	2014	Korea	Postsurgical scar	Split‐scar RCT	14	FCO_2_	PDL	NR	NR	VSS
Monotherapy vs. monotherapy	Manuskiatti et al. [22]	2013	Thailand	Acne scar	Split‐face RCT	24	Er:YAG	FCO_2_	2 sessions	6 months	Clinical improvement, satisfaction
Monotherapy vs. monotherapy	Asilian et al. [23]	2011	Iran	Acne scar	Parallel RCT	64	FCO_2_	Nd:YAG QS	NR	6 months	Clinical improvement
Monotherapy vs. monotherapy	Cho et al. [24]	2010	Korea	Acne scar	Split‐face, evaluator‐blinded RCT	20	NAFL	FCO_2_	1 session	3 months	Improvement, erythema, pain
Monotherapy vs. monotherapy	Tierney et al. [25]	2009	USA	Postsurgical scar	Split‐scar RCT	12	NAFL	PDL	NR	2 months	Improvement percentage
Monotherapy vs. monotherapy	Lee et al. [26]	2009	Korea	Acne scar	Split‐face RCT	18	PDL	Nd:YAG LP	4 sessions	NR	ECCA, satisfaction
Monotherapy vs. monotherapy	Yaghmai et al. [27]	2005	USA	Acne scar	RCT	12	Nd:YAG LP	Nd:YAG 1320	3 sessions	6 months	Improvement percentage
Monotherapy vs. monotherapy	Tanzi and Alster [28]	2004	USA	Acne scar	Prospective controlled trial	20	Diode	Nd:YAG 1320	3 sessions	12 months	Roughness, satisfaction
Monotherapy vs. monotherapy	Elsaie et al. [29]	2018	Egypt	Acne scar	Parallel RCT	58	FCO_2_	NAFL	4 sessions	3 months	Improvement grade
Monotherapy vs. monotherapy	Osman et al. [30]	2024	Egypt	Mixed/postsurgical scar	Parallel RCT	32	Er:YAG	FCO_2_	NR	3 months	VSS subscales

Abbreviations: AFL, ablative fractional laser; ECCA, Echelle d'Evaluation Clinique des Cicatrices d'Acne; FCO_2_, fractional CO_2_ laser; GER, Global Evaluation Response; IGA, Investigator's Global Assessment; LP, long‐pulse; NAFL, non‐ablative fractional laser; NR, not reported in the current extraction sheet; PDL, pulsed dye laser; PIH, post‐inflammatory hyperpigmentation; QS, Q‐switched; VSS, Vancouver Scar Scale.

The network incorporated 10 distinct laser monotherapy nodes—fractional CO_2_ (FCO_2_, 10 600 nm), ablative fractional laser (AFL), fractional Er:YAG (2940 nm), pulsed dye laser (PDL, 585/595 nm), long‐pulse Nd:YAG (1064 nm), Q‐switched Nd:YAG (1064 nm), fractional picosecond Nd:YAG (1064 nm with microlens array or diffractive optical element), non‐ablative fractional laser (NAFL, 1540–1565 nm erbium‐glass), diode laser (1450 nm), and Nd:YAG at 1320 nm—alongside 4 combination nodes representing dual‐laser regimens: PDL combined with NAFL, PDL combined with FCO_2_, ablative fractional laser combined with NAFL, and a combined 585/1064 nm dual‐wavelength protocol. Fractional CO_2_ functioned as the most heavily studied node, appearing in 15 direct comparisons, followed by NAFL (9 comparisons), PDL (6 comparisons), picosecond Nd:YAG (5 comparisons), and Er:YAG and long‐pulse Nd:YAG (4 comparisons each).

### 3.3 Risk of Bias

RoB 2.0 yielded four studies at low overall risk and 22 with some concerns; no study was judged high risk. D1 was low risk in 25/26 studies, with Cohen and Geronemus [2] rated as some concerns because randomization details were insufficiently described. D2 was low risk in six studies and some concerns in 20. D3 was rated as some concerns in all 26 studies. D4 was low risk in 10 studies with clearly blinded outcome assessment and some concerns in 16. D5 was some concerns in all studies because no prospective public analysis plan was available. Full judgments are shown in Table 2 and Figure 2.

**TABLE 2:** Study‐level Cochrane RoB 2.0 assessment.

Study	D1 Randomization	D2 Deviations	D3 Missing data	D4 Outcome measurement	D5 Selective reporting	Overall
Cohen 2016 [2]	Some concerns	Some concerns	Some concerns	Some concerns	Some concerns	Some concerns
Safra 2019 [7]	Low	Some concerns	Some concerns	Some concerns	Some concerns	Some concerns
Kim 2009 [8]	Low	Some concerns	Some concerns	Some concerns	Some concerns	Some concerns
Zhou 2024 [1]	Low	Some concerns	Some concerns	Some concerns	Some concerns	Some concerns
Min 2009 [9]	Low	Some concerns	Some concerns	Low	Some concerns	Some concerns
Liu 2025 [10]	Low	Low	Some concerns	Low	Some concerns	Some concerns
Yuan 2025 [11]	Low	Some concerns	Some concerns	Some concerns	Some concerns	Some concerns
Dai 2023 [12]	Low	Some concerns	Some concerns	Low	Some concerns	Some concerns
Sabry 2022 [13]	Low	Low	Some concerns	Low	Some concerns	Low
Al‐Dhalimi 2021 [14]	Low	Some concerns	Some concerns	Some concerns	Some concerns	Some concerns
Cheng 2021 [15]	Low	Low	Some concerns	Low	Some concerns	Low
Shi 2021 [16]	Low	Low	Some concerns	Low	Some concerns	Low
Sirithanabadeekul 2021 [17]	Low	Some concerns	Some concerns	Some concerns	Some concerns	Some concerns
Kwon 2020 [18]	Low	Some concerns	Some concerns	Low	Some concerns	Some concerns
Chitgopeker 2020 [19]	Low	Low	Some concerns	Low	Some concerns	Low
Shin 2014 [20]	Low	Some concerns	Some concerns	Some concerns	Some concerns	Some concerns
Kim 2014 [21]	Low	Some concerns	Some concerns	Some concerns	Some concerns	Some concerns
Manuskiatti 2013 [22]	Low	Some concerns	Some concerns	Low	Some concerns	Some concerns
Asilian 2011 [23]	Low	Some concerns	Some concerns	Some concerns	Some concerns	Some concerns
Cho 2010 [24]	Low	Low	Some concerns	Some concerns	Some concerns	Some concerns
Tierney 2009 [25]	Low	Some concerns	Some concerns	Low	Some concerns	Some concerns
Lee 2009 [26]	Low	Some concerns	Some concerns	Some concerns	Some concerns	Some concerns
Yaghmai 2005 [27]	Low	Some concerns	Some concerns	Some concerns	Some concerns	Some concerns
Tanzi 2004 [28]	Low	Some concerns	Some concerns	Some concerns	Some concerns	Some concerns
Elsaie 2018 [29]	Low	Some concerns	Some concerns	Some concerns	Some concerns	Some concerns
Osman 2024 [30]	Low	Some concerns	Some concerns	Some concerns	Some concerns	Some concerns

*Note:* D1, bias arising from the randomization process; D2, bias due to deviations from intended interventions; D3, bias due to missing outcome data; D4, bias in measurement of the outcome; D5, bias in selection of the reported result.

**FIGURE 2:**
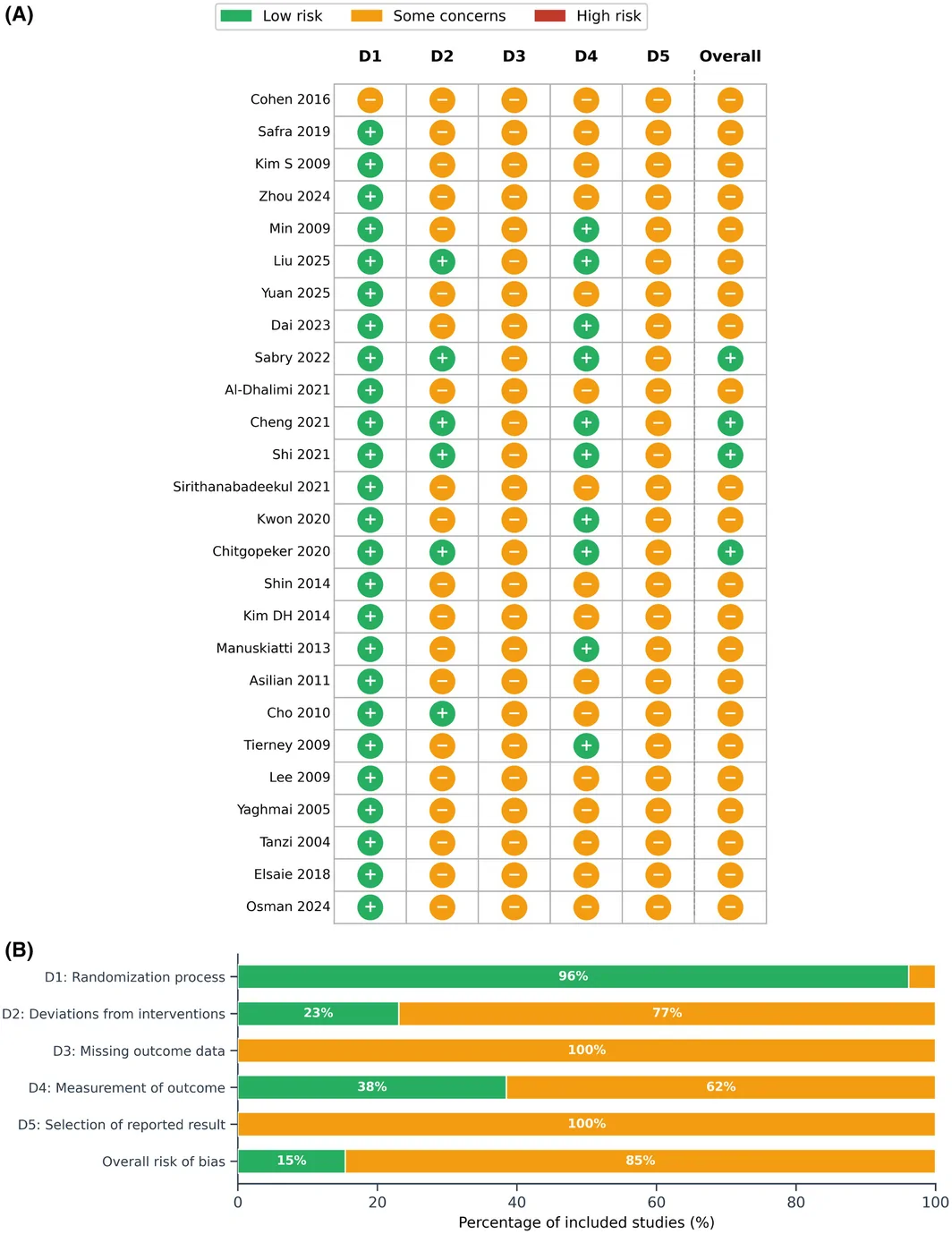
Cochrane RoB 2.0 risk of bias assessment. (A) Study‐level traffic light plot across the five Cochrane RoB 2.0 domains plus overall judgment for all 26 included RCTs. (B) Stacked bar summary showing the proportion of studies rated as low risk, some concerns, or high risk in each domain. Four studies (15%) were rated overall low risk; 22 (85%) were rated as some concerns; none were rated as high risk.

### 3.4 Network Geometry and Evidence Gap Mapping

The geometry of the treatment network revealed a pronounced asymmetry between the depth of evidence supporting monotherapy comparisons and the scarcity of randomized data addressing combined‐laser regimens (Figure 3). Among the 25 studies contributing to the network, 21 (84%) compared two single‐laser modalities against each other, generating a densely interconnected subnetwork with multiple closed loops suitable for consistency testing—the FCO_2_–picosecond–NAFL triangle alone was informed by 10 independent RCTs, and the FCO_2_–Er:YAG–Nd:YAG LP triangle by 3. By contrast, the 4 combination nodes were each supported by a single RCT; the five trials that contributed at least one combination‐versus‐monotherapy comparison enrolled a pooled sample of 172 participants, with no individual combination regimen evaluated in more than one RCT—less than one‐quarter of the evidence base available for FCO_2_ monotherapy alone.

**FIGURE 3:**
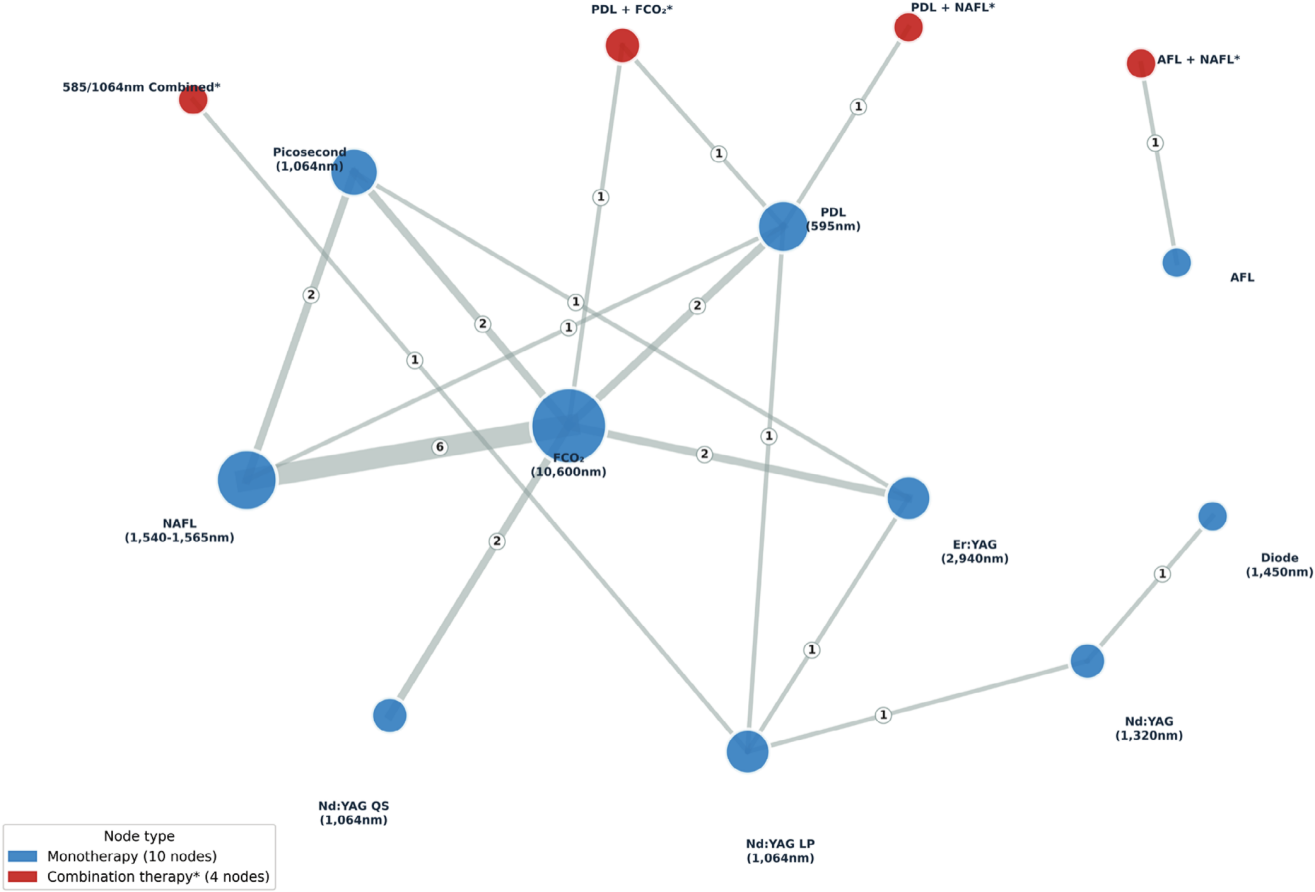
Network of laser treatment comparisons. Network plot of all laser treatment nodes in the analysis. Node size is proportional to the number of studies contributing to each treatment; edge width is proportional to the number of direct head‐to‐head RCTs for each comparison. Blue nodes denote monotherapy (*n* = 10); red nodes (asterisked) denote combination regimens (*n* = 4).

The evidence gap heatmap (Figure 4) quantified this imbalance at the level of individual treatment pairings. Of the 91 theoretically possible pairwise comparisons among the 14 network nodes, direct randomized evidence existed for only 17 (19%), leaving 74 comparisons (81%) entirely devoid of head‐to‐head trial data. Among monotherapy‐versus‐monotherapy pairings, the most thoroughly investigated comparison was FCO_2_ versus NAFL, informed by 6 separate RCTs enrolling a combined 282 participants; FCO_2_ versus picosecond and several other FCO_2_‐anchored contrasts were each supported by 2 RCTs. By contrast, no direct evidence whatsoever was available for clinically relevant monotherapy pairings such as PDL versus Er:YAG, picosecond versus diode, or Q‐switched Nd:YAG versus any combination regimen. Within the combination domain, each of the four included dual‐laser protocols—PDL plus NAFL, PDL plus FCO_2_, AFL plus NAFL, and the 585/1064 nm combined system—had been evaluated in only one RCT apiece, and none of these combination regimens had been compared against each other, thereby precluding any within‐combination ranking.

**FIGURE 4:**
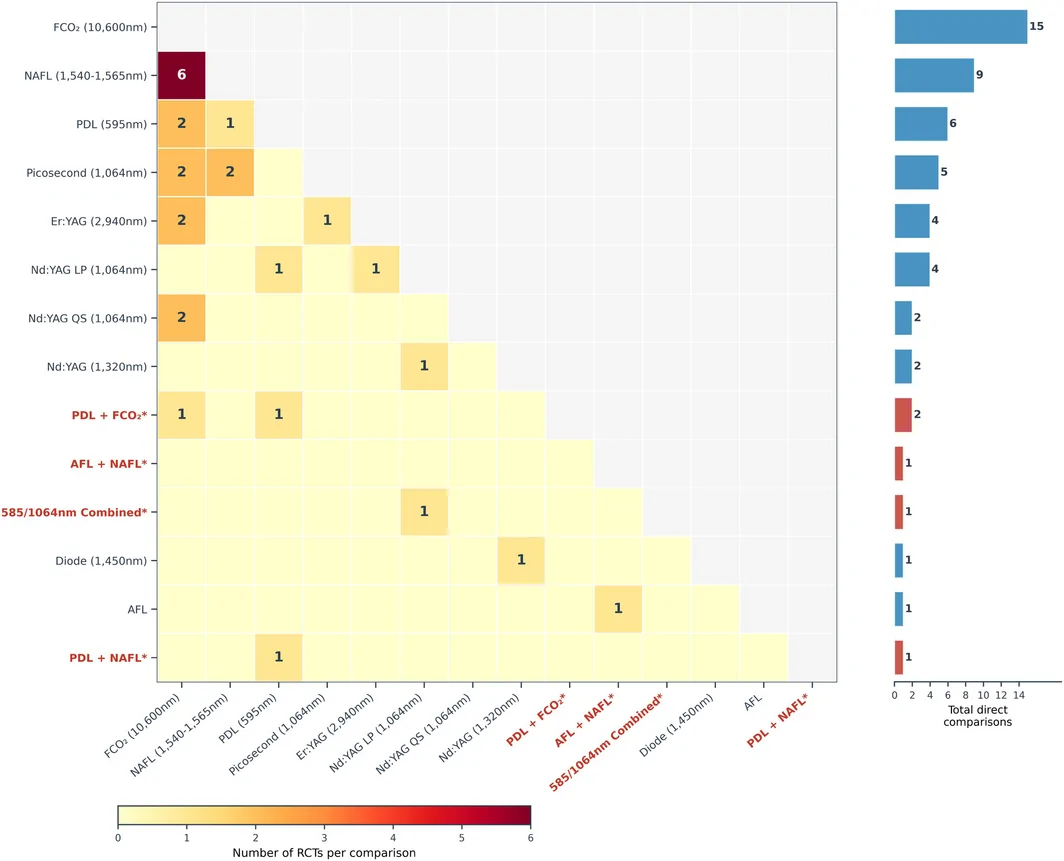
Evidence gap map: Direct RCT comparisons between laser modalities. Evidence gap heatmap showing the density of direct randomized evidence between pairs of laser modalities. Rows and columns are ordered by the total number of direct comparisons (most‐studied modality first). The horizontal bar chart on the left indicates the cumulative number of direct comparisons per node. Empty cells represent complete evidence voids—comparisons for which no head‐to‐head RCT exists. 17 of 91 possible pairwise comparisons (19%) are supported by at least one RCT; 81% constitute evidence gaps.

### 3.5 Combined Laser Versus Monotherapy Outcomes

The four RCTs that directly compared a combined‐laser regimen with a single‐laser modality yielded a consistent directional signal favoring combination treatment, although inter‐study heterogeneity in outcome measures limited formal meta‐analytic pooling across all four. Zhou et al. [1], in the largest of these trials (*n* = 90, parallel‐group design), reported that the PDL‐plus‐NAFL (1565 nm) combination produced significantly greater improvement in Goodman–Baron quantitative scar scores than NAFL monotherapy after a single treatment session, with post‐treatment mean scores of 4.98 ± 2.44 versus 6.67 ± 3.46 (*p* < 0.05), corresponding to a standardized mean difference of approximately 0.56—a medium effect size according to conventional benchmarks. The proportion of patients achieving a one‐to‐two‐grade improvement was 91.1% in the combination arm compared with a notably lower rate in the monotherapy arm, and erythema reduction on chromameter assessment was comparable between groups.

Cohen and Geronemus [2], in a four‐arm prospective trial enrolling 25 patients with post‐Mohs surgical scars, observed that the combination of PDL with fractional CO_2_ ablative resurfacing yielded superior Vancouver Scar Scale and Global Evaluation Response improvements relative to either modality alone, with the combination arm achieving the highest satisfaction scores, although the small per‐arm sample size (*n* = 6–7) precluded definitive statistical comparison. Kim and Cho [8], treating 20 Asian patients with Fitzpatrick skin types IV–V in a split‐face RCT, found that the combination of an ablative fractional laser at low fluence with a non‐ablative resurfacing laser outperformed high‐fluence ablative fractional monotherapy in overall scar appearance while producing fewer complications, including reduced erythema duration and a lower incidence of post‐inflammatory hyperpigmentation. Min et al. [9], comparing a combined 585/1064 nm dual‐wavelength laser system with long‐pulse Nd:YAG monotherapy in 19 patients, documented significant ECCA score reductions on both facial halves with no statistically significant intermodality difference overall, though the combined system demonstrated superiority for deep boxcar scars specifically, and histological analysis revealed significantly greater collagen deposition on the combination‐treated side.

### 3.6 Monotherapy Comparisons and Network Ranking

Among the 25 RCTs that defined the connected network, the 15 studies providing extractable effect‐size estimates drove the quantitative pooling and *P*‐score treatment rankings, with FCO_2_ serving as the reference comparator in the main connected network; the remaining 10 network‐eligible trials contributed to the evidence‐gap analysis and the characterization of network geometry (Figure 4) but not to the numerical effect estimates (Figures 5 and 6). Fractional CO_2_ occupied a central position in the network and served as the common comparator in the majority of included trials, facilitating both direct and indirect estimation of relative treatment effects.

**FIGURE 5:**
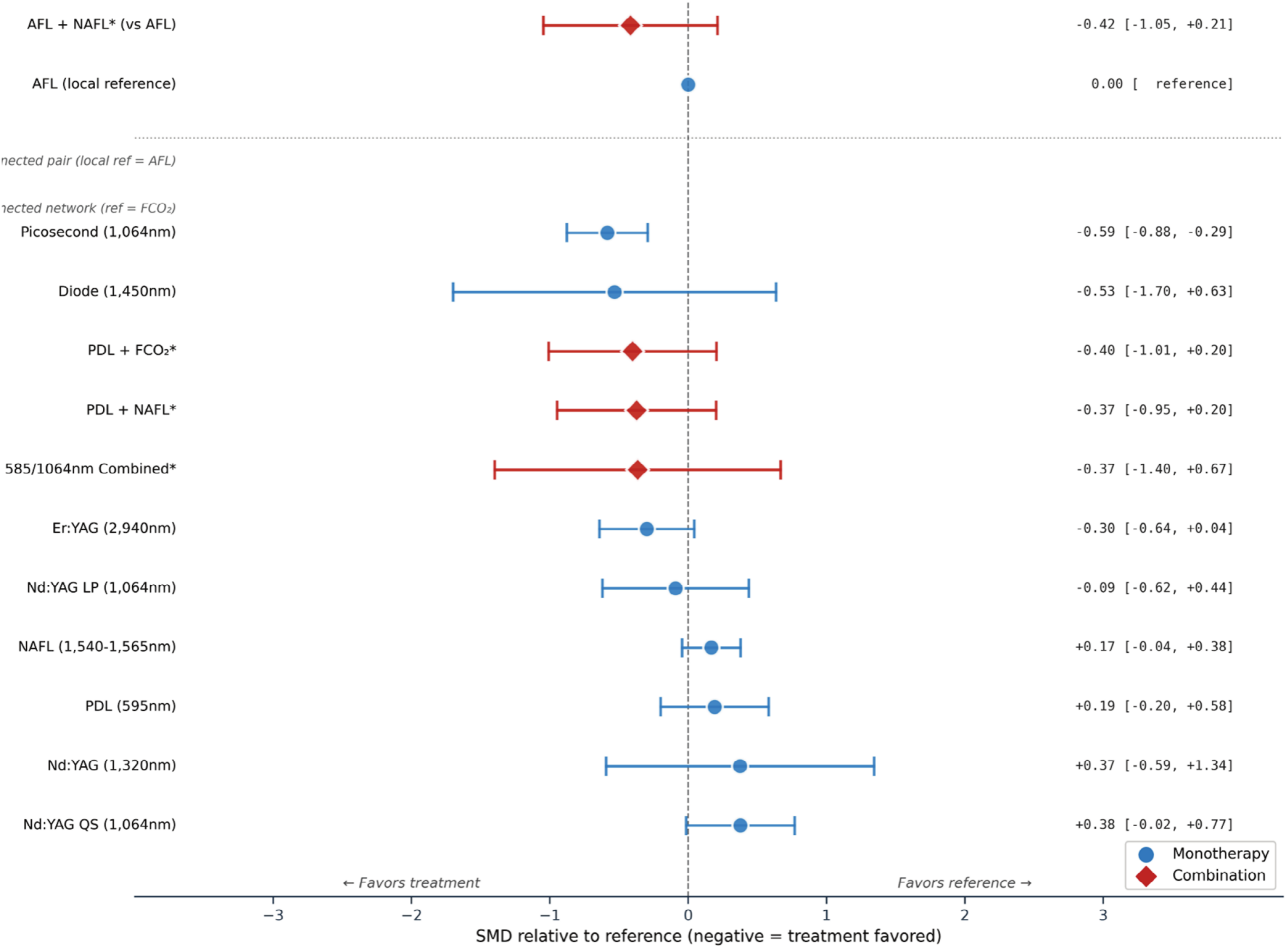
Forest plot: Treatments vs. fractional CO_2_ (main network reference). Random‐effects network meta‐analysis forest plot showing standardized mean differences (SMD) of each laser treatment versus fractional CO_2_ as the reference in the main connected network. Circles indicate monotherapy; diamonds indicate combination therapy. AFL and AFL + NAFL form a disconnected subnetwork and are shown separately with AFL as the local reference. Effect estimates are derived from a contrast‐based generalized least‐squares NMA model.

**FIGURE 6:**
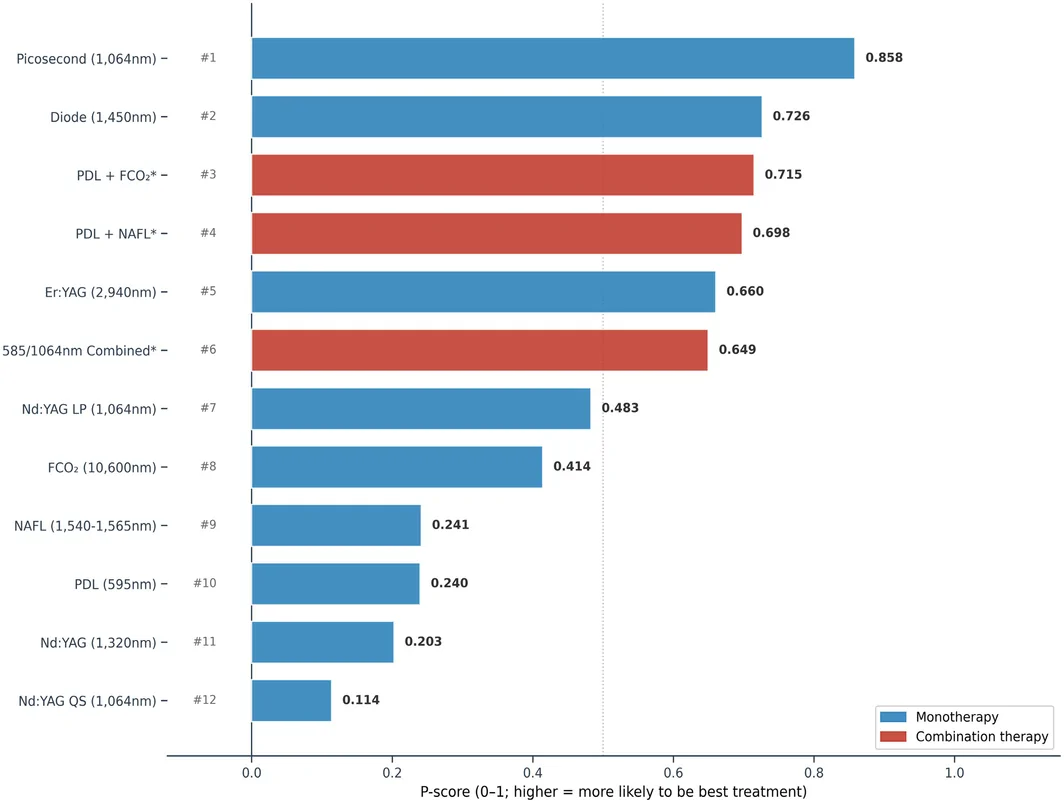
*P*‐score treatment ranking—main connected network. Treatment ranking based on *P*‐scores (frequentist analogue of SUCRA), calculated within the main connected network of 12 laser nodes. Higher *P*‐score indicates higher probability of being the most effective treatment. Blue bars denote monotherapy; red bars denote combination therapy. AFL/AFL + NAFL are excluded from ranking because they form a disconnected pair.

The comparison between fractional CO_2_ and non‐ablative fractional lasers, the most data‐rich contrast in the network, was addressed by 6 RCTs encompassing 282 participants. The pooled evidence indicated that FCO_2_ produced marginally greater clinical improvement than NAFL across most scar grading instruments, although several individual studies—including the large split‐scar trial by Chitgopeker et al. [19] (*n* = 100)—reported no significant difference in patient‐rated satisfaction or long‐term scar appearance between the two modalities. Shin et al. [20] noted that while clinical improvement grades were comparable between AFL and NAFL for post‐thyroidectomy scars (2.45 ± 0.99 vs. 2.35 ± 0.85, *p* = 0.752), the two modalities demonstrated complementary strengths: AFL was superior in reducing scar hardness (*p* = 0.026), whereas NAFL achieved greater improvement in erythema (*p* = 0.035) and pigmentation (*p* = 0.003). Elsaie et al. [29], in the only parallel‐group RCT to compare these modalities (*n* = 58), reported higher mean improvement grades with FCO_2_ but without reaching statistical significance (*p* = 0.1).

Picosecond Nd:YAG lasers occupied the highest *P*‐score position (0.858) in the main connected network, with a pooled standardized mean difference of −0.59 (95% CI −0.88 to −0.29) relative to fractional CO_2_ (Figures 5 and 6). The direct evidence supporting this contrast, however, comprises only two RCTs that themselves yield discrepant conclusions: Sirithanabadeekul et al. [17] reported a significantly greater ECCA score reduction with picosecond treatment (*p* = 0.007) whereas Yuan et al. [11] found no statistically significant difference (*p* = 0.209). When appraised within the GRADE framework for network meta‐analyses using the CINeMA tool, the certainty of evidence for this comparison is rated as very low, reflecting the limited number of direct trials, the uniform “some concerns” risk‐of‐bias ratings across all picosecond studies, the absence of prospective protocol registration addressing potential reporting bias, and the inconsistency between the two directly contributing estimates. The network‐derived efficacy signal, together with a consistently more favorable safety profile observed across procedure‐related pain, erythema duration, crusting, and post‐inflammatory hyperpigmentation, is therefore interpreted as a robust but provisional indication that picosecond Nd:YAG warrants further investigation as a preferred monotherapy option rather than as an established first‐line choice.

Dai et al. [12], in a separate comparison against ablative fractional Er:YAG rather than FCO_2_, reported equivalent ECCA percentage reduction between picosecond with microlens array and Er:YAG (39.11% vs. 43.73%, *p* > 0.05), alongside significantly lower pain scores and a shorter duration of crusting on the picosecond‐treated side. In comparisons with NAFL, both Shi et al. [16] and Kwon et al. [18] observed superior efficacy of picosecond lasers in improving atrophic acne scars—particularly V‐type and U‐type subtypes—with objective skin‐flatness measurements confirming the clinical impression.

Fractional Er:YAG and fractional CO_2_ were compared directly in 3 RCTs. Manuskiatti et al. [22] found no significant difference in investigator‐assessed clinical improvement between the two ablative modalities (*p* = 0.90) despite significantly higher patient satisfaction with CO_2_ treatment (5.8 ± 2.0 vs. 3.2 ± 1.4, *p* < 0.001) in a cohort of 24 Asian patients. Osman et al. [30], evaluating immature and mature postsurgical scars, reported that Er:YAG achieved significantly lower Vancouver Scar Scale scores than CO_2_ across all four subscale domains—vascularity (0.71 ± 0.76 vs. 1.67 ± 0.71, *p* = 0.021), pigmentation (0.43 ± 0.53 vs. 1.22 ± 0.83, *p* = 0.046), pliability (0.43 ± 0.53 vs. 1.44 ± 0.73, *p* = 0.008), and height (0.57 ± 0.79 vs. 1.89 ± 1.27, *p* = 0.031)—although patient satisfaction and quality‐of‐life indices did not differ between groups.

The diode (1450 nm) and Nd:YAG (1320 nm) lasers, representing older non‐ablative platforms, were compared in the sole trial by Tanzi and Alster [28], which demonstrated greater roughness‐average improvement with the diode laser at 6 months (−26.14 μm vs. −9.89 μm, *p* = 0.005), paralleled by higher patient satisfaction scores (5.7 vs. 4.6 on a 10‐point scale), notwithstanding comparable blinded clinical assessment grades between the two devices (1.81 vs. 1.67).

### 3.7 Subgroup Analysis by Scar Etiology

Stratification of the included studies by scar type revealed a marked imbalance: 19 of 26 trials (73%) enrolled patients with atrophic acne scars exclusively, whereas only 7 addressed postsurgical or post‐traumatic scars. Within the acne scar subgroup, the network retained all 10 monotherapy nodes and 3 of the 4 combination nodes, preserving network connectivity and permitting indirect comparisons across the full range of laser platforms. The postsurgical scar subgroup, by contrast, was limited to 7 studies spanning 5 treatment nodes (FCO_2_, PDL, NAFL, PDL plus FCO_2_, and AFL plus NAFL), with insufficient closed loops to support a separate network meta‐analysis; the results for this subgroup are therefore presented as a qualitative synthesis. Among the postsurgical scar studies, both the Kim et al. [21] and Tierney et al. [25] split‐scar trials suggested comparable efficacy between vascular‐targeting lasers (PDL) and resurfacing lasers (FCO_2_ or NAFL) for linear surgical scars, with PDL showing an advantage in reducing scar vascularity and NAFL or FCO_2_ producing greater textural improvement.

### 3.8 Sensitivity Analysis

Restriction of the network to the 4 trials rated as having a low overall risk of bias—all of which employed blinded outcome assessment—did not materially alter the relative treatment rankings, though the limited number of retained comparisons reduced the precision of all effect estimates substantially. Exclusion of the 4 combination nodes and analysis of the monotherapy subnetwork alone preserved all major treatment‐ranking patterns, confirming that the overall network structure was not unduly influenced by the sparsely populated combination nodes. Global consistency between direct and indirect evidence was assessed using the design‐by‐treatment interaction model and did not reveal statistically significant inconsistency across the network (*p* > 0.05). Local consistency was evaluated by node‐splitting across the closed loops within the main connected network; no individual comparison yielded a statistically significant difference between its direct and indirect effect estimates (all *p* > 0.05), supporting the plausibility of the transitivity assumption underpinning the indirect comparisons.

### 3.9 Publication Bias Assessment

Visual inspection of the comparison‐adjusted funnel plot (Figure 7) did not reveal marked asymmetry suggestive of systematic small‐study effects, although the limited number of studies per comparison—particularly for the combination nodes—constrained the statistical power of this assessment. The absence of funnel‐plot asymmetry should be interpreted with caution given that only 17 of the 91 possible pairwise comparisons in the network were informed by direct evidence, and that the preponderance of split‐face designs with small sample sizes inherently limits the sensitivity of funnel‐plot‐based bias detection methods.

**FIGURE 7:**
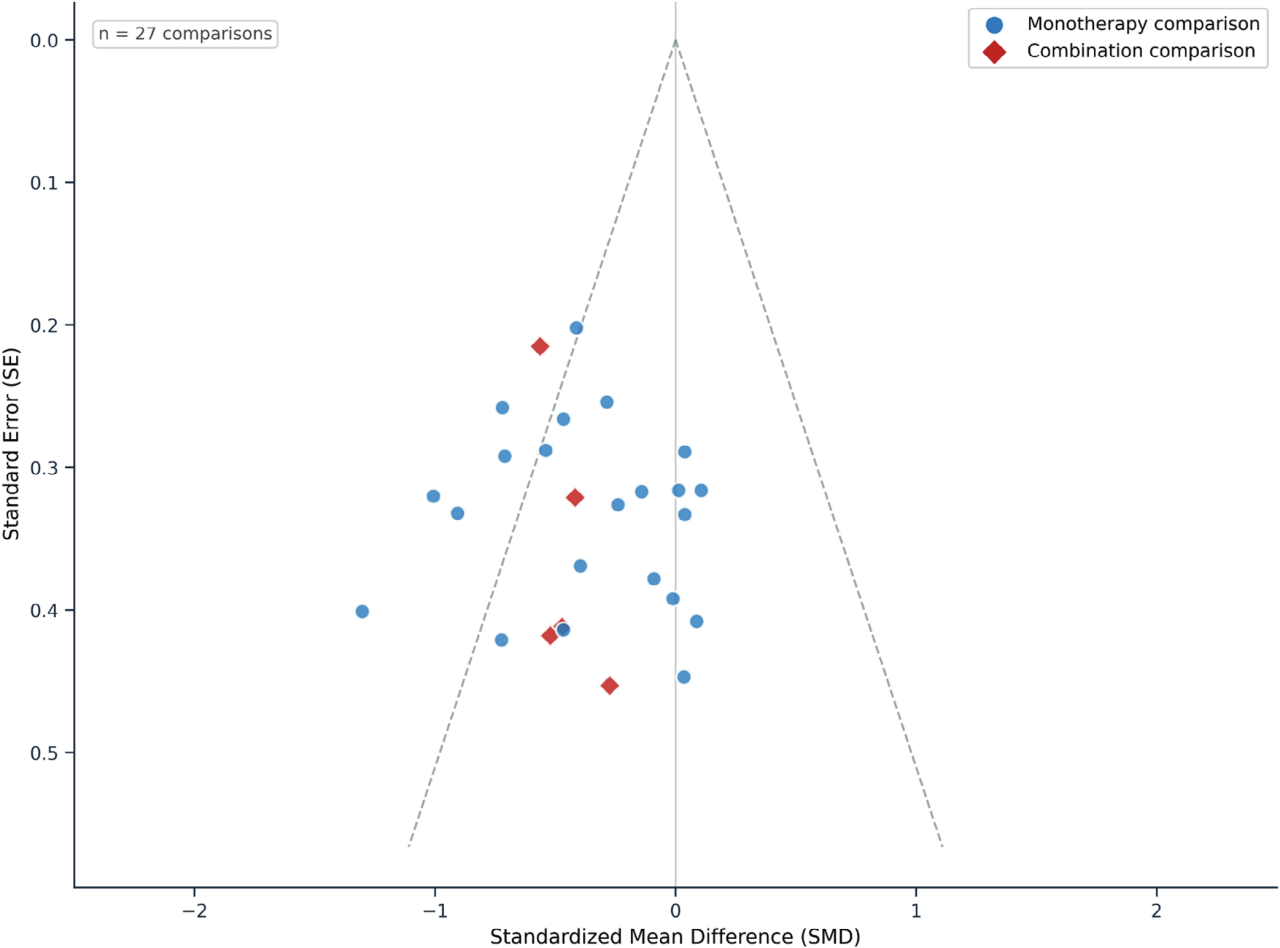
Comparison‐adjusted funnel plot. Comparison‐adjusted funnel plot for visual assessment of small‐study effects and potential publication bias. Each point represents a single pairwise comparison (*n* = 27); dashed lines indicate pseudo 95% confidence limits.

## 4 Discussion

The central finding of this systematic review and network meta‐analysis is not a definitive treatment ranking but rather a structural revelation: the randomized evidence base for laser‐based scar treatment, whereas substantial in aggregate, is distributed in a profoundly uneven manner that leaves the majority of clinically relevant comparisons—and virtually all questions about combined‐laser efficacy—without direct experimental support. Of the 91 theoretically possible pairwise contrasts among the 14 laser nodes identified in this review, only 17 were informed by at least one RCT, and the four combination regimens that constitute the most rapidly evolving frontier of clinical practice were each supported by a single trial, none of which compared one combination against another. This evidence architecture has immediate practical consequences: clinicians who adopt dual‐laser protocols do so on the basis of mechanistic plausibility and uncontrolled case series rather than the comparative effectiveness data that would be expected for any other therapeutic domain at a similar stage of clinical adoption.

The directional consistency observed across the four combination‐versus‐monotherapy RCTs—each favoring the dual‐laser arm, albeit with varying effect magnitudes and outcome instruments—provides a biologically coherent signal that merits cautious interpretation. Zhou et al. [1] demonstrated a medium‐sized standardized effect (SMD ≈ 0.56) favoring PDL combined with 1565 nm NAFL over NAFL monotherapy for Goodman–Baron scar scores, a finding consistent with the hypothesis that coupling vascular‐selective photothermolysis with fractional collagen remodeling addresses complementary dimensions of scar pathology that neither modality alone can optimize. The mechanistic rationale for this synergy is well articulated in the dermatologic laser literature: PDL targets oxyhemoglobin within the proliferative neovasculature of immature scars and has been shown to downregulate transforming growth factor‐β expression through heat‐shock protein 70‐mediated pathways, whereas non‐ablative fractional lasers create microthermal zones of subepidermal coagulation that trigger a wound‐healing cascade culminating in Type III collagen replacement by Type I collagen over ensuing months. The observation by Kim and Cho [8] that combining a low‐fluence ablative fractional pass with a non‐ablative laser achieved superior cosmetic outcomes with fewer complications than high‐fluence ablative monotherapy suggests that combination protocols may permit clinicians to reduce per‐modality fluence settings and thereby mitigate the dose‐dependent adverse‐event profile—particularly post‐inflammatory hyperpigmentation in Fitzpatrick skin types IV–V—without sacrificing therapeutic efficacy.

These provisional observations, however encouraging, must be weighed against the methodological fragility of the underlying evidence. The pooled sample size across the five combination‐versus‐monotherapy RCTs was 172 participants, a figure that would be considered inadequate to detect a moderate treatment effect in a single adequately powered trial, and the heterogeneity of outcome instruments employed—Goodman–Baron grades, Vancouver Scar Scale, ECCA, and investigator‐rated quartile scales—precluded formal meta‐analytic pooling across all four studies. The absence of any head‐to‐head comparison between different combination regimens means that the present review cannot address the question of whether PDL‐plus‐FCO_2_ is superior to, equivalent to, or inferior to PDL‐plus‐NAFL or AFL‐plus‐NAFL—a gap that represents perhaps the single most consequential evidence void for clinical decision‐making in multimodal laser scar management.

The monotherapy subnetwork, by contrast, offered a considerably richer evidence base from which to draw provisional inferences. Fractional CO_2_ emerged as the de facto reference standard by virtue of its ubiquity in the trial literature rather than by demonstrated superiority, featuring in 13 of 25 network comparisons and serving as one arm of the five best‐populated direct contrasts. The finding that FCO_2_ and NAFL produce broadly comparable long‐term clinical improvement despite marked differences in downtime and acute adverse‐event profiles carries practical significance for treatment individualization: patients with darker skin phototypes or those unwilling to accept the 7‐ to 14‐day recovery associated with ablative resurfacing may be counseled that non‐ablative alternatives, whereas potentially requiring a greater number of sessions, are likely to yield similar endpoint scar appearance. The complementary domain‐specific advantages documented by Shin et al. [20]—ablative treatment excelling in pliability and scar height reduction, non‐ablative treatment showing superiority for erythema and pigmentation—provide a mechanistic foundation for the very combination strategies whose evidence base this review has found to be so sparse, and suggest that future combination trials pairing these two modality classes could exploit well‐characterized complementary tissue effects.

Picosecond Nd:YAG lasers, a relatively recent addition to the scar treatment armamentarium, demonstrated a distinctive profile across the seven RCTs in which they were evaluated: comparable or superior clinical efficacy to established ablative fractional platforms, coupled with a consistently lower burden of procedure‐related pain, post‐treatment erythema, crusting duration, and PIH incidence. The photomechanical mechanism through which picosecond pulses induce laser‐induced optical breakdown—generating intracutaneous cavitation vacuoles that stimulate cytokine release and collagen remodeling without the thermal coagulation zones produced by longer‐pulse devices—may account for this favorable safety‐to‐efficacy ratio.

Given the very low certainty attached to the current network estimate for the picosecond‐versus‐fractional‐CO_2_ contrast within the CINeMA framework, the preliminary efficacy signal documented here warrants confirmation in adequately powered head‐to‐head trials before picosecond platforms can be positioned as a preferred monotherapy for atrophic acne scars; if such confirmation is obtained, the combination of a favorable efficacy signal with the consistently lower procedure‐related pain, post‐treatment erythema, crusting duration, and post‐inflammatory hyperpigmentation incidence documented across the seven picosecond RCTs may support preferential adoption for pigment‐prone skin phototypes and position these platforms as attractive combination partners for vascular‐targeting lasers in future multimodal protocols.

The comparison between the two ablative fractional platforms—Er:YAG and CO_2_—yielded a nuanced pattern that resists simple hierarchical ranking. Investigator‐rated global improvement was indistinguishable between the two modalities in the Manuskiatti et al. [22] split‐face trial, yet the same study reported significantly higher patient satisfaction with CO_2_ treatment, an apparent paradox that may reflect differences in the trajectory of recovery and visible tissue response between the two wavelengths rather than differences in final scar quality. The contrasting findings of Osman et al. [30], in which Er:YAG demonstrated superiority across all four VSS subscales in postsurgical scars, highlight the potential for scar etiology and maturity to modify the relative performance of ablative platforms—a hypothesis that warrants dedicated investigation in stratified or factorial trial designs.

Placing these findings within the context of the existing NMA literature illuminates both the contribution and the boundaries of the present analysis. The Zhao et al. [3] Bayesian NMA, which incorporated non‐laser interventions such as PRP and radiofrequency into its network, ranked fractional CO_2_ plus PRP as the highest‐performing regimen—a conclusion that, whereas potentially valid within its broader comparator framework, is not actionable for the clinician whose treatment options are restricted to laser devices. By excluding all non‐laser nodes, the present review sacrificed network breadth for clinical specificity, accepting a sparser network as the necessary cost of answering a focused question. The finding that 81% of possible laser‐versus‐laser comparisons lack any direct RCT evidence would have been invisible within the mixed‐modality framework of prior NMAs, in which the missing laser‐versus‐laser comparisons were obscured by the presence of non‐laser bridging nodes. In this sense, the evidence‐gap characterization reported here is not merely a byproduct of the analytical approach but a primary deliverable: it renders visible a structural deficiency in the trial landscape that has direct implications for the design and prioritization of future research.

This review has several limitations. The search did not include Embase, Web of Science, or regional databases. Most trials were small split‐face studies; laser parameters were heterogeneous, and outcome measures were not standardized. Only 15 of 25 network‐eligible trials contributed quantitative effect sizes, whereas 10 informed network geometry only, and Safra et al. [7] remained disconnected because its comparator was no treatment. These factors may have introduced imprecision and availability bias into the rankings.

The evidence‐gap map generated by this review lends itself to a concrete research prioritization framework. The most urgent evidentiary need is a multicenter, adequately powered RCT comparing at least two distinct combined‐laser protocols against each other and against their component monotherapies in a factorial or multi‐arm design—a study that would, in a single enrollment effort, populate multiple currently vacant cells in the comparison matrix and dramatically improve the precision of the network ranking. A standardized core outcome set for laser scar trials, incorporating both clinician‐rated instruments such as ECCA or POSAS and patient‐reported outcome measures, would reduce the heterogeneity that currently impedes cross‐study synthesis and maximize the cumulative value of future trials. Given the concentration of existing evidence in Fitzpatrick skin types II–IV, dedicated investigation in skin types V–VI—populations at highest risk for PIH and for whom the safety‐to‐efficacy trade‐off is most consequential—represents a further priority that the current evidence base does not adequately address. Although early single‐device data suggest that fractional erbium lasers can be used safely in skin types IV–VI [31], no comparative trial has evaluated combination protocols specifically in these populations.

## 5 Conclusion

This laser‐only systematic review and network meta‐analysis identified 26 RCTs across 14 laser nodes and showed that direct evidence is concentrated in a small subset of comparisons. Picosecond Nd:YAG achieved the highest exploratory *P*‐score in the main monotherapy network, but certainty was very low. Combined‐laser evidence was limited to 5 small RCTs evaluating 4 distinct regimens, with no head‐to‐head combination trials. Because 81% of possible contrasts lacked direct randomized evidence, treatment selection still depends heavily on mechanistic reasoning and individual experience, underscoring the need for adequately powered comparative trials.

## Author Contributions

Q.Y. conceived and designed the study, developed the review protocol, interpreted the findings, obtained funding, and drafted and revised the manuscript. J.W. contributed to literature searching, study screening, data extraction, and manuscript drafting. K.Y. contributed to risk‐of‐bias assessment, data verification, and interpretation of the network meta‐analysis. G.F. contributed to methodological guidance, statistical interpretation, and critical revision of the manuscript. Y.L. contributed to data extraction, evidence gap mapping, and preparation of tables and figures. C.L. contributed to statistical analysis, network meta‐analysis implementation, and visualization of results. Y.L. contributed to study screening, verification of extracted outcomes, and Supporting Information preparation. J.S. contributed to reference checking, quality control, and manuscript revision. L.G. contributed to clinical interpretation of laser treatment modalities and critical review of the manuscript. All authors read and approved the final manuscript.

## Funding

This work was supported by Beijing Jishuitan Research Funding (code: LC202504): A clinical comparative study on the treatment of early scars with pulsed dye laser (PDL), ultra‐pulsed fractional carbon dioxide laser (UFCL), and their combination.

## Ethics Statement

Ethical approval was not required because this study is a systematic review and network meta‐analysis based exclusively on previously published data. No patient‐identifiable information was accessed or used.

## Conflicts of Interest

The authors declare no conflicts of interest.

## Supporting information


**Appendix S1:** Database‐specific search strategies.

## Data Availability

The data extracted and analyzed during the current study are available from the corresponding author upon reasonable request.
