# Supplementary material for: Navigating Combined Laser Modalities Versus Monotherapy for Atrophic Acne and Postsurgical Scar Improvement: A Systematic Review and Network Meta‐Analysis
Source: J Cosmet Dermatol. 2026 Oct 29;25(11):e71230. doi: 10.1111/jocd.71230 (PMC13720220; doi:10.1111/jocd.71230)
Supplement: Supplementary file 1 [file JOCD-25-e71230-s001.docx]

**Supplementary Material S1**

Database-specific search strategies

**PubMed (via MEDLINE)**

*From inception through April 2026*

#1 "Laser Therapy"[MeSH] OR "Lasers"[MeSH] OR "Laser Therapy"[Pharmacological Action]
#2 laser*[tiab] OR "laser therapy"[tiab] OR "laser treatment"[tiab] OR "laser resurfacing"[tiab]
#3 "fractional CO2"[tiab] OR "fractional carbon dioxide"[tiab] OR "CO2 laser"[tiab] OR "carbon dioxide laser"[tiab] OR FCO2[tiab]
#4 "Er:YAG"[tiab] OR "erbium:YAG"[tiab] OR "erbium laser"[tiab] OR "erbium-doped"[tiab]
#5 "pulsed dye laser"[tiab] OR PDL[tiab] OR "595 nm"[tiab] OR "585 nm"[tiab]
#6 "Nd:YAG"[tiab] OR "neodymium:YAG"[tiab] OR "neodymium laser"[tiab] OR "1064 nm"[tiab]
#7 "picosecond laser"[tiab] OR picosecond[tiab] OR "pico laser"[tiab] OR PicoSure[tiab] OR PicoWay[tiab]
#8 "thulium laser"[tiab] OR thulium[tiab] OR "1927 nm"[tiab]
#9 "diode laser"[tiab] OR "810 nm"[tiab] OR "940 nm"[tiab]
#10 "non-ablative fractional"[tiab] OR "nonablative fractional"[tiab] OR NAFL[tiab] OR "1540 nm"[tiab] OR "1550 nm"[tiab] OR "erbium glass"[tiab]
#11 "ablative fractional"[tiab] OR AFL[tiab] OR "ablative laser"[tiab]
#12 #1 OR #2 OR #3 OR #4 OR #5 OR #6 OR #7 OR #8 OR #9 OR #10 OR #11

#13 "Cicatrix"[MeSH]
#14 scar*[tiab] OR cicatri*[tiab]
#15 "acne scar"[tiab] OR "acne scars"[tiab] OR "acne scarring"[tiab] OR "post-acne scar"[tiab] OR "postacne scar"[tiab]
#16 "atrophic scar"[tiab] OR "atrophic scars"[tiab] OR "ice pick"[tiab] OR icepick[tiab] OR boxcar[tiab] OR "rolling scar"[tiab] OR "rolling scars"[tiab]
#17 "postsurgical scar"[tiab] OR "postoperative scar"[tiab] OR "surgical scar"[tiab] OR "post-surgical scar"[tiab] OR "postoperative scars"[tiab] OR "surgical scars"[tiab]
#18 "Acne Vulgaris"[MeSH]
#19 #13 OR #14 OR #15 OR #16 OR #17 OR #18

#20 "Randomized Controlled Trial"[pt] OR "Controlled Clinical Trial"[pt]
#21 random*[tiab] OR randomis*[tiab] OR randomiz*[tiab] OR RCT[tiab]
#22 "controlled trial"[tiab] OR "clinical trial"[tiab]
#23 "split-face"[tiab] OR "half-face"[tiab] OR "split-body"[tiab] OR "split-scar"[tiab] OR "intraindividual"[tiab]
#24 #20 OR #21 OR #22 OR #23

#25 #12 AND #19 AND #24

**Europe PMC**

*From inception through April 2026*

(
TITLE_ABS:"laser therapy" OR TITLE_ABS:laser* OR TITLE_ABS:"laser resurfacing" OR
TITLE_ABS:"fractional CO2" OR TITLE_ABS:"fractional carbon dioxide" OR TITLE_ABS:"CO2 laser" OR
TITLE_ABS:"Er:YAG" OR TITLE_ABS:"erbium laser" OR TITLE_ABS:"pulsed dye laser" OR
TITLE_ABS:"Nd:YAG" OR TITLE_ABS:"neodymium laser" OR TITLE_ABS:"picosecond laser" OR
TITLE_ABS:picosecond OR TITLE_ABS:"thulium laser" OR TITLE_ABS:"diode laser" OR
TITLE_ABS:"non-ablative fractional" OR TITLE_ABS:"nonablative fractional" OR
TITLE_ABS:"ablative fractional"
)
AND
(
TITLE_ABS:scar* OR TITLE_ABS:cicatri* OR TITLE_ABS:"acne scar*" OR TITLE_ABS:"post-acne scar*" OR
TITLE_ABS:"atrophic scar*" OR TITLE_ABS:"ice pick" OR TITLE_ABS:boxcar OR TITLE_ABS:"rolling scar*" OR
TITLE_ABS:"postsurgical scar*" OR TITLE_ABS:"postoperative scar*" OR TITLE_ABS:"surgical scar*" OR
TITLE_ABS:"Acne Vulgaris"
)
AND
(
TITLE_ABS:random* OR TITLE_ABS:RCT OR TITLE_ABS:"controlled trial" OR TITLE_ABS:"clinical trial" OR
TITLE_ABS:"split-face" OR TITLE_ABS:"half-face" OR TITLE_ABS:"split-scar" OR TITLE_ABS:intraindividual
)

**OpenAlex**

*From inception through April 2026*

search=(
laser OR "laser therapy" OR "laser resurfacing" OR "fractional CO2" OR "carbon dioxide laser" OR
"Er:YAG" OR "pulsed dye laser" OR "Nd:YAG" OR picosecond OR "thulium laser" OR
"diode laser" OR "non-ablative fractional" OR "nonablative fractional" OR "ablative fractional"
)
AND (
scar OR cicatrix OR "acne scar" OR "post-acne scar" OR "atrophic scar" OR "ice pick" OR
boxcar OR "rolling scar" OR "postsurgical scar" OR "postoperative scar" OR "surgical scar"
)
AND (
randomized OR randomised OR trial OR RCT OR "split-face" OR "half-face" OR "split-scar" OR intraindividual
)

**ClinicalTrials.gov**

*From inception through April 2026*

Condition or disease:
scar OR "acne scar" OR "atrophic scar" OR "surgical scar"

Intervention/treatment:
laser

Study type:
Interventional (Clinical Trial)
